# Supplementary figures and images for: Field Prevalence and Pathological Features of Edwardsiella tarda Infection in Farmed American Bullfrogs (Aquarana catesbeiana)
Source: Animals (Basel). 2025 Aug 25;15(17):2487. doi: 10.3390/ani15172487 (PMC12427314; doi:10.3390/ani15172487)

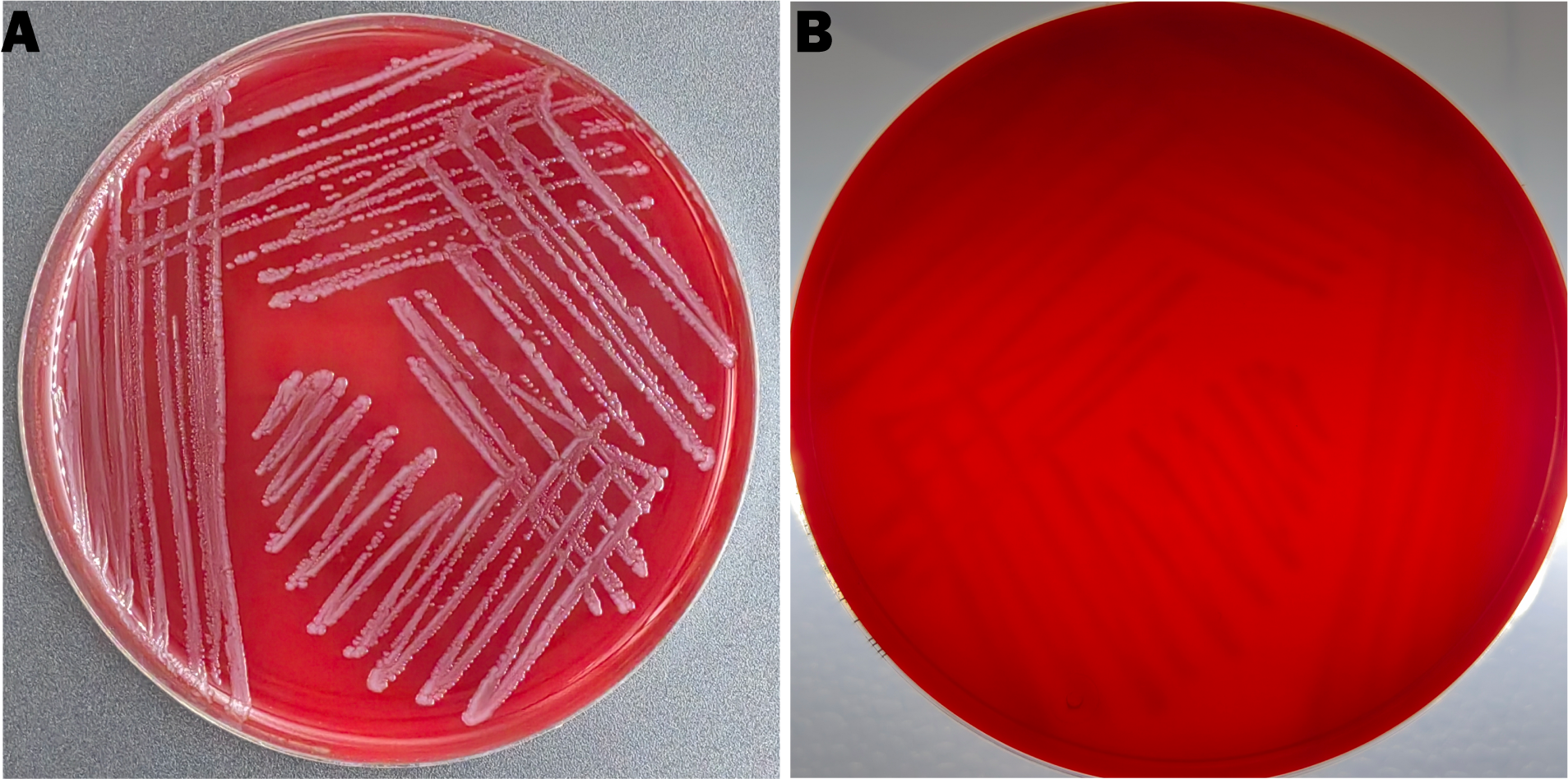

Supplement: Supplementary file 1 [file animals-15-02487-s001.zip › FigureS1.tif]
